# How cunning is the puppet-master? Cestode-infected fish appear generally fearless

**DOI:** 10.1007/s00436-022-07470-2

**Published:** 2022-03-21

**Authors:** P. Andreas Svensson, Ramin Eghbal, Ramona Eriksson, Emelie Nilsson

**Affiliations:** Department of Biology and Environmental Science, Linnaeus University, 39231 Kalmar, Sweden

**Keywords:** Host-parasite interaction, Behavioural manipulation, Nutrient theft, Odour, Baltic Sea, PITT

## Abstract

Trophically transmitted parasites have life cycles that require the infected host to be eaten by the correct type of predator. Such parasites should benefit from an ability to suppress the host’s fear of predators, but if the manipulation is imprecise the consequence may be increased predation by non-hosts, to the detriment of the parasite. Three-spined sticklebacks (*Gasterosteus aculeatus*) infected by the cestode *Schistocephalus solidus* express reduced antipredator behaviours, but it is unknown whether this is an example of a highly precise manipulation, a more general manipulation, or if it can even be attributed to mere side effects of disease. In a series of experiments, we investigated several behaviours of infected and uninfected sticklebacks. As expected, they had weak responses to simulated predatory attacks compared to uninfected fish. However, our results suggest that the parasite induced a general fearlessness, rather than a precise manipulation aimed at the correct predators (birds). Infected fish had reduced responses also when attacked from the side and when exposed to odour from a fish predator, which is a “dead-end” for this parasite. We also tested whether the reduced anti-predator behaviours were mere symptoms of a decreased overall vigour, or due to parasite-induced hunger, but we found no support for these ideas. We propose that even imprecise manipulations of anti-predator behaviours may benefit parasites, for example, if other behaviours are altered in a way that increases the exposure to the correct predator.

**Supplementary Information:**

The online version contains supplementary material available at 10.1007/s00436-022-07470-2.

## Introduction

Parasites that require successive hosts to complete their life cycle are fascinating organisms of great interest to evolutionary biologists (Cezilly et al. 2010; Hughes et al. 2012). A successful transfer from one host to another is critical to the parasite’s fitness, but the probability of this occurring is often low. Many classes of parasites rely on tropical transmission, that is, one host must be caught and consumed by another host in order for the parasite to survive and reproduce. Parasites are expected to evolve unique adaptations to push the odds of successful transmission in their favour. One possibility is to manipulate the host so that it starts behaving in a way that benefits the parasite. Tales of advanced “mind control” are popular, not only among parasitologists and evolutionary ecologists but also to the general public (Moore 2002; Zimmer 2000). The idea of parasite puppet-masters is fascinating, both for the biological complexity, and for the fundamental questions that are raised, such as the blending of phenotypes, and the issue of free will (Hughes et al. 2012).

The tape-worm *Schistocephalus solidus,* Steenstrup, 1857 is a model organism for studies of parasite-induced behavioural change. Its eggs are excreted with bird faeces into water, and after hatching, the free swimming coracidium stage is eaten by a copepod, which is the first intermediate host and inside of which it develops into a procercoid (Smyth 1962). If the infected copepod is eaten by a three-spined stickleback (*Gasterosteus aculeatus*, L), the procercoid can develop into a plerocercoid inside the abdominal cavity of the fish (Smyth 1962). If the infected stickleback, in turn, is eaten by a susceptible final host, typically a piscivorous bird, *S. solidus* may, at last, develop into an adult, reproduce and complete its life cycle.

*S. solidus* are known to manipulate their copepod hosts in seemingly adaptive ways (Franz and Kurtz 2002; Hafer and Milinski 2015; Hammerschmidt et al. 2009; Weinreich et al. 2013). For example, the presence of a procercoid *increases* the copepod’s fear of potential predators early in the infection, but *reduces* the fear of attack after reaching the stage where it is infective to sticklebacks (Urdal et al. 1995). As a consequence, the likelihood of being consumed by the second host is at first supressed and then enhanced, which indicates an adaptive manipulation (Weinreich et al. 2013). Also the second intermediate host, the three-spined stickleback, is known to express reduced antipredator behaviours when infected by *S. solidus*, at least after the point where the plerocercoid has reached the size necessary to infect a bird (Barber et al. 2004; Tierney and Crompton 1992). Infected sticklebacks act bolder and have a reduced response to predators (Giles 1983; Milinski 1985). Thus, one can argue that *S. solidus* also manipulates its stickleback host in order to increase the probability of completing its complex life cycle. However, caution should be taken before assigning adaptive explanations, and the case for active host manipulation in cestode parasites may have been overstated (Poulin 2000). If infection leads to changes in behaviour, there are several other possible explanations to consider: the changes can be coincidentally caused by the struggle between host and parasite, it may actually be an adaptive host response, or it may be a non-adaptive side effect of the pathology of the infection (Poulin 1994).

A suggested explanation for the altered phenotype of infected sticklebacks is that a growing plerocercoid will drain the energetic reserves of the fish, which has far-reaching effects, such as reduced reproductive investment and altered behaviours (Heins and Baker 2008; Heins et al. 2010a; Heins and Brown-Peterson 2010; Schultz et al. 2006). One proposal is that the reduced anti-predator responses are only symptoms of a more general loss of vigour caused by the parasite’s nutrient theft (Cezilly et al. 2010). In contrast, Hafer and Milinski (2016) suggested that the nutrient theft of *S. solidus* should lead to higher activity and boldness in infected sticklebacks. They reasoned that the energetic drain of a growing parasite makes infected fish experience an increased state of hunger. Rather than inducing lethargy, this should drive them to become bolder foragers (Hafer and Milinski 2016).

Poulin (1995) suggested that a key prediction for the manipulation hypothesis is that the behavioural change needs to increase risk of predation by the *correct* predator. Anti-predation manipulation with such high level of precision is known from several parasite-host systems. For example, both infected amphipods (*Gammarus roeseli*, Gervais 1835) and snails (*Potamopyrgus antipodarum*, Gray 1843) have been shown to behave in ways that should increase the predation from suitable bird hosts but reduce non-host predation by fishes (Levri 1998; Medoc et al. 2009). In rainbow trout infected by *Diplostomum sp.,* the changes in anti-predator behaviour expose them specifically to avian predation (correct hosts) while responses to fish predators (non-hosts) are unaffected (Seppälä et al. 2012). *S. solidus* coracidia can infect many species of copepods and the plerocercoid can infect many species of birds, but the procercoid stage specifically infects three-spined sticklebacks (Henrich et al. 2013), and these two species share a long evolutionary history (Nishimura et al. 2011). The strict host specificity and the long co-evolution increase the likelihood that *S. solidus* has evolved mechanisms to affect stickleback behaviour, not just by debilitating its physiology but also by advanced manipulation, for example, by targeting the sticklebacks central nervous system (Fredensborg 2014; Hebert et al. 2017). As non-host predation should constitute a strong negative selection pressure, authors have speculated that precise manipulations of host behaviours, such as those preferentially exposing them to the correct predators, should be common (Barber et al. 2000). It is possible that *S. solidus* has had the time to evolve a behavioural manipulation that increases the stickleback’s susceptibility to bird predation while reducing the risk of fish predation. The prediction here is that infected stickleback should respond to the threat of fish predation in the same way as uninfected individuals.

On the other hand, some authors have questioned the need for parasites to evolve highly precise manipulations (Milinski 1985; Parker et al. 2009). Such adaptations may be costly, and parasites should not invest in them if the ecology of the host makes them unnecessary (Poulin 1994). Even imprecise manipulation can be adaptive if, for example, infected prey encounter the correct predators more often than dead-end predators (Barber and Huntingford 1995; Parker et al. 2009). Barber and Huntingford (1995) proposed that infected sticklebacks may suffer increased predation also from non-host predators, but attempts to test this prediction have either been indirect or used non-comparable experimental treatments. For example, examinations of the gut contents of salmon revealed that these predatory fish seemed to target sticklebacks infected with *S. solidus* (Jakobsen et al. 1988). Ness and Foster (1999) compared the responses of sticklebacks that were attacked either by a model tern in the laboratory, or by a preserved trout in the field, and concluded that infected sticklebacks had reduced responses to both, and may therefore suffer increased predation also by dead-end fish predators. No attempt has yet been made to test the responses of sticklebacks to two comparable threat stimuli, or to a stimulus that is unequivocally from a dead-end predator.

There are important regional differences in how sticklebacks respond to *S. solidus* infection. For example, susceptibility to infection varies greatly between distant stickleback populations (Kalbe et al. 2016) and in some areas infected sticklebacks will suffer supressed reproduction, but not in others (Heins and Baker 2008; Macnab et al. 2009; Schultz et al. 2006). In addition, sticklebacks have evolved counter-adaptations to *S. solidus* in certain populations but not others (Heins and Baker 2014; Heins et al. 2014; Lohman et al. 2017; Weber et al. 2017). It is therefore important to expand investigations of this model system to new areas and to a wider range of environments (Barber 2013; Macnab et al. 2009). Poulin and Maure (2015) argued for more empirical work on host manipulation by parasites, and that they should also be done in previously unstudied populations. The Kalmar Sound area of the Baltic Sea is such a place, with a high density of three-spined sticklebacks with, at least locally, a very high *S. solidus* prevalence (see results). Sticklebacks from this area have, to our knowledge, not previously been studied with respect to behavioural effect of *S. solidus* infections.

We designed a series of experiments to investigate the behaviours of Baltic Sea sticklebacks infected by *S. solidus*. We hypothesized that naturally infected fish would express reduced responses to simulated predatory attacks, but not in situations simulating fish predation. We also predicted that behavioural effects of infection were not merely consequences of a general lethargy, nor that they could not be explained by greater hunger in infected sticklebacks.

## Materials and methods

### Fish capture and husbandry

Three-spined sticklebacks for use in the experiments were collected with a beach seine in shallow water (0–1 m depth) in the spring of 2014 (simulated attack, foraging and exploration experiments) and 2020 (perch odour experiment) at beaches in Kalmar in the Baltic Sea. After transport to the nearby laboratory, fish were held in groups in 60-L holding aquaria with flow-through brackish (7 psu) water from the Baltic Sea. The maximum time fish were kept in the holding tanks was two weeks. The temperature followed the outside ambient temperature and varied between 8 and 14 °C. The light regime was set at 8/16 h dark/light. Fish were fed *Daphnia magna,* small pieces of herring and white worm daily. Sticklebacks vary in belly roundness and females with mature gonads may have greatly distended bellies similar to individuals with heavy infection. Thus, we could only determine which sticklebacks carried *S. solidus* plerocercoids after dissection (see below). The behavioural trials were therefore blind in this regard. After finding a high parasite prevalence and very few multiple infections (see results), we also made a collection of sticklebacks by setting fine gill nets 200 m from the shore at 3 m depth in May 2014. From this, a randomly chosen subset of sticklebacks were euthanized and screened for *S. solidus* infections.

### Responses to simulated attacks

A 1 × 1 m test arena was filled with 7 psu sea water to 15 cm (Fig. 1 in Supplementary Material). In each corner, a plastic plant was placed as shelter, and a clear cylinder (Ø15 cm) was positioned in the middle of the arena. A single stickleback was placed in the cylinder, and when it had resumed normal swimming behaviour (i.e. pectoral sculling, typically after 0–5 min) the cylinder was lifted by a pulley system. The fish was then exposed to one of two stimuli, operated from behind a curtain. The “overhead attack” consisted of a black hardboard plate (21 × 25 cm) dropping from a height of 94 cm to 5 cm above the water surface. The “lateral attack” consisted of a black hardboard plate with folding-out sides that quickly approached the side of the arena. The two stimuli were designed so that the apparent change in size (as seen by the fish), as well as the speed of the approach, would be similar. Depending on the exact position of the focal fish, the perceived angle of both stimuli expanded from approximately 15° to 60°. Each fish was exposed to both types of attack in a randomized order. The behavioural responses of 56 sticklebacks were recorded from immediately before to 10 min after the attack using two video cameras; one that focussed on the central area and one with an overview of the entire arena. We classified the immediate behavioural responses into two categories. 1) “No response”, when the fish either remained still or continued to swim slowly without pausing. 2) “Escape”, when the fish either made a straight dash, or a staggered swim, away from the centre of the arena. We also quantified the time it took for the fish to reach cover and the time it took for them to recover (i.e. to resume normal swimming behaviour: pectoral sculling) after the attack. Ten minutes after the first attack, the fish was moved back to the cylinder, and when it had resumed normal swimming behaviour it was released and attacked again, using the alternate stimulus.

### Response to perch odour

In order to test the responses to a non-host predator, we exposed sticklebacks to perch odour. We chose to quantify spine erection as this is a sensitive measure of fear and vigilance in sticklebacks (Landeira-Dabarca et al. 2019). It is also a behaviour that should be unaffected by the bulk of the parasite, which can affect swimming performance of infected individuals (Jolles et al. 2020). The experimental set-up was modified from (Landeira-Dabarca et al. 2019). Ahead of the trials, one of three 20–30 cm TL Eurasian perch (*Perca fluviatilis*, chosen at random) was moved from their holding basin to a 25 l container with fresh sea water where they remained for 2–3 h. Water from this container was used for the predator odour treatment. Individual sticklebacks were moved from their holding tanks to a 30L test arena (Fig. 2 in Supplementary Material). These were covered on three sides with dark plastic and contained fresh sea water, an air stone, gravel on the bottom and rocks and artificial vegetation for shelter. After 10 min acclimation, the trial began by adding 2 dl of water through a hose connected to the test arena. This was either from a container with fresh sea water (control), or from the perch container (predator odour treatment). For the next five minutes, the experimenter observed the stickleback through a slit in a screen in front of the arena. The amount of time that the stickleback had its dorsal spines fully erected was logged with stop watches. Forty individuals were tested twice, once with control water and once with predator odour. The treatment order was alternated between test subjects. The test arenas were cleaned carefully and left to dry before changing treatment.

### Effects of infection and hunger on foraging and exploration

We quantified foraging and exploration behaviours in sticklebacks that were either infected or uninfected by *S. solidus*, and that had either been starved for one day, or had been allowed to feed on *D. magna* until satiation just prior to the trials. We used a 20 × 37 cm arena to test the behavioural trade-off between foraging and remaining in shelter (Fig. 3 in Supplementary Material). The sheltered area contained plastic plants and was covered with black fabric on the sides and over the top. The other (“risky”) side of the arena was uncovered and had a spotlight placed directly above. In this illuminated area, we placed a 20 ml glass vial containing ~ 50 live *D. magna*. Fifty sticklebacks were randomly selected for this experiment, half of which had recently been fed (satiated) and half that had not been fed for 24 h (starved). A fish was placed in a clear cylinder at the edge of the shaded area and was left to acclimatise for 5 min. After this, the cylinder was lifted with a pulley system. We video recorded for 15 min and noted the time until the fish had left the sheltered area and attempted to eat the prey by striking at the vial. Later same day (time between experiments was 1–4 h) we also measured the propensity to explore an unfamiliar environment using a simple labyrinth (20 × 37 cm, Fig. 4 in Supplementary Material) using the same 50 fish as in the foraging experiment (satiated and starved). This labyrinth had three plastic plants for shelter. A fish was placed in a clear cylinder inside the start area and was left to acclimatise for 5 min. After this, the fish was released, and the behaviour was video recorded for 15 min. We quantified the time until the fish had left the start area and until it had reached the goal area (i.e. explored the entire labyrinth).

### Dissection

After behavioural trials, the fish were euthanized with an overdose of benzocaine and dissected in order to determine the presence of any *S. solidus* parasites. This was done for the 146 fish used in the behavioural experiments, for 19 fish used in pilot studies as well as the 125 fish caught in the second field collection made 200 m from shore (total n = 290). It was noted whether the fish were infected with *S. solidus* (other types of parasites were not investigated in this study) and the weight and number of all plerocercoids. We also noted the sex of the fish.

### Statistics

Statistics were performed in R 4.0.2. The percentages of time with erect spine were arcsine square-root transformed and analysed in a linear mixed effects model with “fish identity” as random factor. All time-to-event data were recorded as a bivariate response with a binary component describing whether the behaviour (e.g. escape) occurred or not, and a continuous component describing the time to the event. If the event did not occur (i.e. censored data), the fish was given the maximum time as a ceiling value. To account for the censoring, time-to-event data were analysed with Cox proportional hazards models using the R package Survival. In the predator escape experiment, where individuals were tested once per stimulus, the data were analysed with mixed effects Cox models using the R package coxme, where “fish identity” was entered as a random factor. Model simplification and the significance of interactions and of main effects were based on log-likelihood ratio (LR) tests (*α* = 0.05). If models had marginally significant interactions (0.05 < *P* < 0.2), the two main effects were interpreted both in a new two-way model with the interaction removed and in two separate one-way models after splitting the data.

## Results

### Prevalence estimates

A total of 165 sticklebacks were caught with the beach seine and 117 of these were infected with *S. solidus* plerocercoids (prevalence: 71%). Plerocercoid mass ranged from 0.21–0.88 g (mean ± SD: 0.49 ± 0.11 g), meaning that all *S. solidus* were well above the size of 0.05 g, which is the limit for being infective to birds (Tierney and Crompton 1992; Tierney et al. 1993). The proportion of single infections was 88% among infected fish: only ten fish were infected by two plerocercoids and one fish had three plerocercoids. The sample was balanced with respect to sex (54.8% females, exact binomial test, *p* = 0.22) and sexes did not differ in prevalence (Fisher’s exact test, *p* = 0.15). If a mature and an immature plerocercoid co-infect the same fish they may, theoretically, counteract the effects of each other (Hafer and Milinski 2016). However, as all plerocercoids were well above the size limit for infectivity, we did not expect such conflicts within our fish, and we therefore pooled all infected fish (single and multiple infections) in the analyses. In the experiment with simulated predator attacks, 39 sticklebacks were infected and 17 were uninfected. In the predator odour experiment, 22 were infected and 18 uninfected. In the foraging and exploration experiments, 43 were infected and 7 uninfected. The field collection with gill nets further from the shore gave a total catch of several thousand sticklebacks. From these we randomly selected 125 that were euthanized, dissected and screened for *S. solidus* infections. None of these sticklebacks were infected with *S. solidus*, and the difference in prevalence compared to the in-shore sample was significant (0 vs. 71%, Fisher exact test, *p* < 0.001).

### Responses to simulated attacks

Individual sticklebacks were exposed to simulated predator attacks from above and from the side in a randomised order. Most uninfected sticklebacks responded by escaping: 60% escaped when attacked from the side, and 100% when attacked from above. Among infected fish, 46% escaped when attacked from the side, and 67% escaped when attacked from above. Thus, the overhead attacks elicited stronger responses overall, despite our use of two visually comparable stimuli. The difference in response due to attack type was significantly smaller in infected fish (loglinear glm with Poisson errors, interaction effect (Infection:Attack type:Response type): *ΔAIC* = 3.62, *p* = 0.018). Thus, while sticklebacks generally escaped more often when attacked from above, this difference was smaller in fish infected with *S. solidus*. When the fish were attacked from the side, there was no significant effect of infection on the propensity to escape (Fisher exact test, *p* = 0.56). When the attack came from above, however, infected fish were significantly less likely to escape compared to uninfected fish (Fisher exact test, *p* = 0.005).

**Fig. 1:**
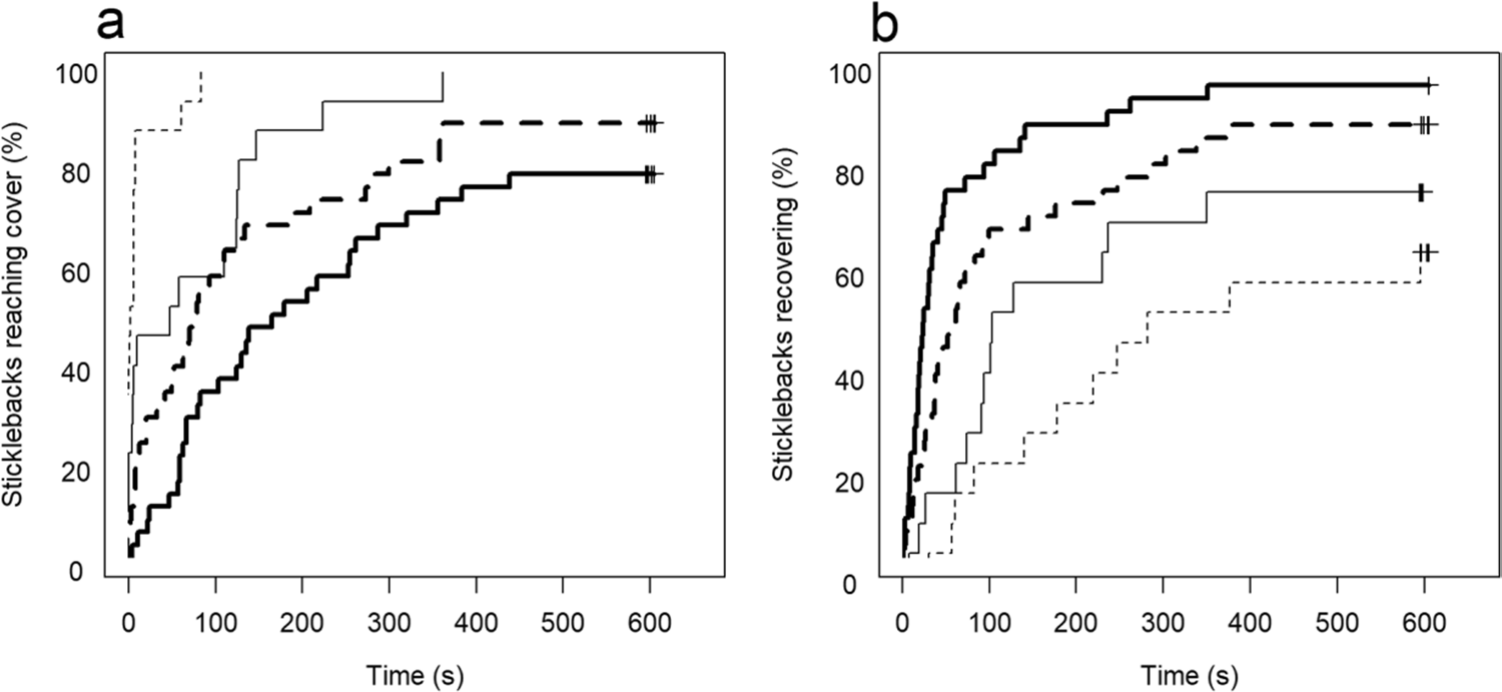
Kaplan–Meier curves of the time from a simulated attack until the focal fish had had reached cover (a), and until it had recovered and resumed normal swimming behaviour (b). Bold lines are sticklebacks infected by *S. solidus*, while thin lines are uninfected. The attacks came either from above (dashed lines) or from the side (solid lines). The plus symbols to the right indicate censored data (i.e. when the event did not occur within the maximum time)

Both infection status and the type of attack affected the time it took for fish to reach cover. When attacked from above, 90% of the uninfected fish dashed straight to cover, while only 20% of infected fish did so (Fig. 1). Both groups reached cover faster if attacked from above than from the side (Cox mixed effects model, LR tests; infected fish: *χ*^*2*^ = 7.18*, d.f.* = 1, *p* = 0.007; uninfected fish: *χ*^*2*^ = 8.99, *d.f.* = 1, *p* = 0.003). However, this difference was marginally smaller in infected fish (interaction effect, Cox mixed effects model, LR test; *χ*^*2*^ = 2.95, *d.f.* = 1, *p* = 0.089). Thus, while both groups responded stronger to the overhead attack, the difference was somewhat reduced in infected fish.

We measured the time from the simulated attack until the sticklebacks had recovered and resumed normal swimming behaviour (pectoral sculling). There was no significant interaction between attack type and infection status on this parameter (Cox mixed effects model, LR test; *χ*^*2*^ = 0.1082, *d.f.* = 1, *p* = 0.74), and the model was re-fitted without this term. Overall, sticklebacks took significantly longer to recover after an overhead attack compared to a lateral attack (LR test; *χ*^*2*^ = 15.25, *d.f.* = 1, *p* = 0.0001, Fig. 1). Infected fish were significantly faster to resume normal swimming compared to uninfected fish (LR test; *χ*^*2*^ = 14.32, *d.f*. = 1, *p* = 0.0002).

### Responses to perch odour

**Fig. 2:**
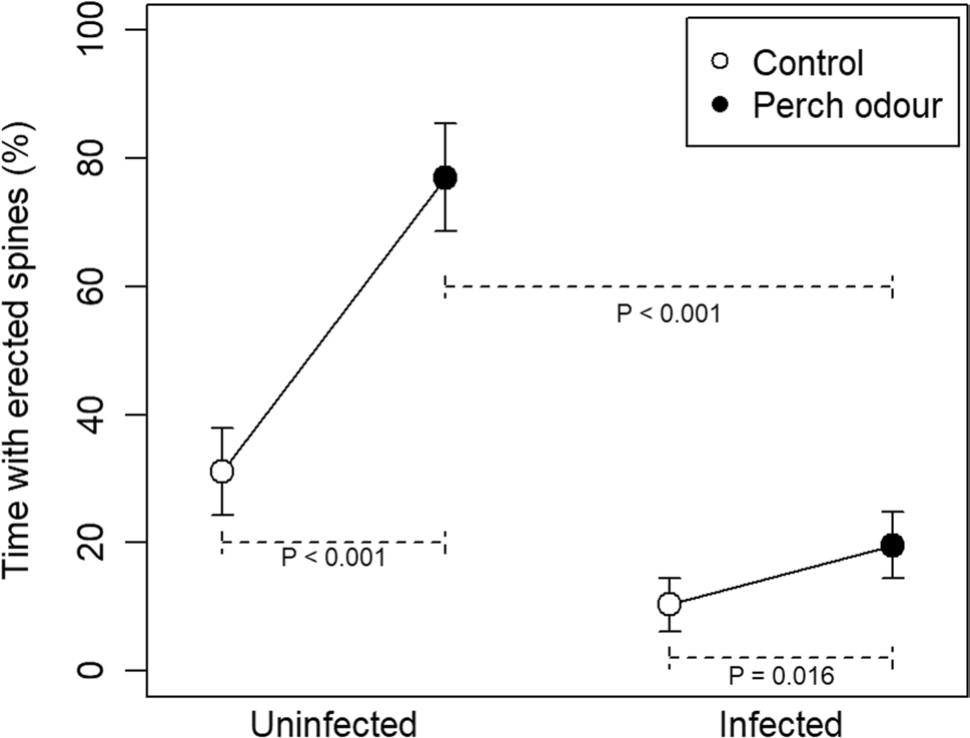
The mean percentage of time (± SE) that three-spined sticklebacks had their dorsal spines erected. Individuals with or without a *S. solidus* infection were tested in both control water and in water with the odour from a live fish predator (an adult perch). P values from linear mixed models

Analysis of the spine erection behaviour revealed a significant interaction effect between infection status and odour treatment (linear mixed model, Treatment:Infection: *t*_38_ = 4.15, *p* < 0.001, Fig. 2). This means that the increase in spine erection in response to perch odour differed between uninfected and infected sticklebacks. To investigate the results further, the analysis was split by infection status. Both infected and uninfected fish increased their spine erection when exposed to perch odour (paired t tests; *p* < 0.001 and *p* = 0.016, respectively). However, uninfected fish had their spines raised more in both situations (t tests; control treatment *p* = 0.006 and odour treatment *p* < 0.001). Thus, infected fish performed less spine erection overall, and although perch odour increased their spine erection significantly, both the change, and the resulting level was lower than in uninfected fish.

### Effects of infection and hunger on foraging and exploration

**Fig. 3:**
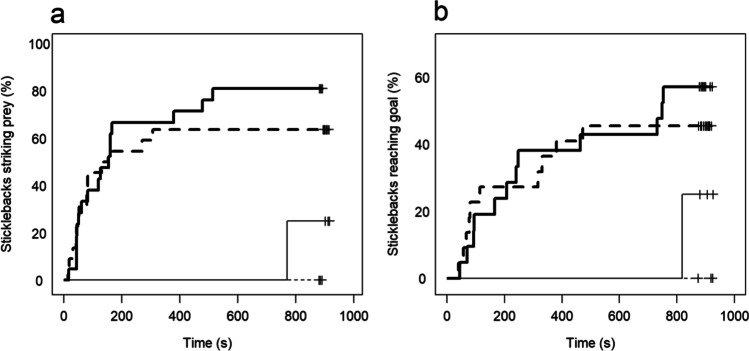
Kaplan–Meier curves of the time until the focal fish first attacked prey placed in a “risky” area (a), and the time until fish had explored a simple labyrinth (b). Bold lines are sticklebacks infected by *S. solidus*, while thin lines are uninfected. The fish were either satiated (solid lines) or starved (dashed lines). The plus symbols to the right indicate censored data (when the event did not occur within the maximum time)

There was no significant interaction between hunger treatment and infection status on the time it took for fish to leave the shelter and attack the prey (log-likelihood reduction of a Cox proportional hazards model: *χ*^*2*^ = 0.89, *d.f.* = 1, *p* = 0.35, Fig. 3). There was also no main effect of hunger treatment (LR test; *χ*^*2*^ = 0.77, *d.f.* = 1, *p* = 0.38) and the model was re-fitted without these terms. The final model showed that infected fish were significantly faster to attack the prey, compared to uninfected fish (Cox proportional hazards model, likelihood ratio test: 9.68, *d.f.* = 1, *p* = 0.002).

There were no significant interactions between hunger treatment and infection status on the time it took fish to i) leave the start area or to ii) reach the goal area (log-likelihood reductions of two Cox proportional hazards models: *χ*^*2*^ < 1.58, *d.f.* = 1, *p* > 0.21). The hunger treatment had no effect on the exploration behaviours (LR tests; leave start area, *d.f.* = 1, *p* = 0.09; reach goal, *d.f.* = 1, *p* = 0.11). However, infected fish were significantly faster to leave the start area (LR test, *d.f*. = 1, *p* = 0.01) and significantly faster to reach the goal (LR test, *d.f.* = 1, *p* = 0.04, Fig. 3) compared to uninfected fish.

## Discussion

Sticklebacks infected by *S. solidus* had weaker responses to simulated predatory attacks compared to uninfected fish, and this included responses to being attacked from the side and to the odour of a fish predator. Our results did therefore not support the idea of a highly precise parasite manipulation favouring the correct predators (birds). We found no support for the idea that reduced anti-predator behaviours are mere symptoms of decreased vigour, or are caused by parasite-induced hunger.

It is well established that sticklebacks infected by *S. solidus* express reduced anti-predator responses (Barber et al. 2004; Giles 1983, 1987a; Ness and Foster 1999; Tierney et al. 1993). This has been proposed to be due to active manipulation by this parasite (Barber et al. 2004; Talarico et al. 2017), but questions remained about the degree of precision with regard to different types of predation threat. A precise manipulation in this context will be if *S. solidus* decreases the sticklebacks’ fear of the correct predators (birds) while maintaining or increasing it for dead-end predators (fish). We designed an experiment to deliver two perceptually comparable stimuli: an object approaching directly from above, simulating bird strike, and an object of the same perceived size approaching from the side. Infected fish were less likely to escape, slower to reach cover and quicker to return to normal swimming behaviour. Although overhead attacks elicited the strongest reactions in both groups, the reduction of anti-predator behaviours in infected fish was most pronounced when attacked from above. When attacked from the side, infected fish behaved more like uninfected fish. This can be seen as support for a precise manipulation of anti-predator behaviours, favouring bird predation. However, given the artificial nature of the two stimuli, we interpret the results with some caution. Quinn and co-workers (2012) pointed out that sticklebacks can be targeted by birds also from the side (e.g. by cormorants and mergansers), and there are in fact many such diving birds in the Kalmar area. A greater susceptibility to lateral attacks may therefore actually be advantageous to *S. solidus*. However, even if laterally attacking predators may sometimes be birds, overhead attacks are unlikely to be from fish. It is therefore interesting that the responses to the stimulus most similar to a bird strike were the ones most strongly reduced by *S. solidus* infection.

To proceed we used a stimulus that unequivocally belongs to a dead-end predator, namely the odour of an adult perch. If an infected stickleback is eaten by a perch it spells disaster for *S. solidus*, and under the precise manipulation hypothesis we expected infected fish to have a retained, or enhanced, response to perch odour. There was a dramatic increase in spine erection when uninfected sticklebacks were exposed to perch odour. Infected sticklebacks also responded, but to a much lesser degree. Studies that have used whole fish as stimuli (live cichlid: Milinski 1985; preserved trout: Ness and Foster 1999) also report that infected sticklebacks have reduced responses to these threats. Taken together, our results support the idea that *S. solidus* manipulate their stickleback host in a general fashion, so that they to some extent also lose fear toward non-host predators.

One alternative to the manipulation explanation is that reduced anti-predator responses in infected individuals are symptoms of a general lethargy caused by the parasites’ nutrient theft (Cezilly et al. 2010). Our results did not support this idea. Although infected sticklebacks appeared sluggish in response to simulated predator attacks, they were otherwise more active: they were faster than uninfected fish to leave shelter and find food, and quicker to explore unknown areas. Our results are supported by a recent study where experimentally infected sticklebacks had reduced anti-predation behaviours, while behaviours such as exploration and foraging were unchanged (Talarico et al. 2017). This was interpreted as evidence against a “systemic impairment” and as support for *S. solidus* specifically manipulating predator avoidance (Talarico et al. 2017).We found that infected sticklebacks not only performed foraging and exploration behaviours on the level of uninfected sticklebacks, but a significantly higher level. A possible explanation for this difference is that Talarico et al. (2017) used laboratory-reared sticklebacks that were habituated to aquaria, while we used recently caught fish from the field. Our uninfected sticklebacks were rather reluctant to explore the aquaria and they had their spines raised around 30% of the time even when undisturbed. Possibly, all our test arenas were perceived as “risky” by the uninfected fish. It may be that studies that use captive sticklebacks habituated to aquaria underestimate the effects *S. solidus* infection on boldness as it manifests in nature, where there is great spatial complexity and a pervasive threat of predation.

Previous studies support our results that *S. solidus* infection will increase foraging and risk-taking in sticklebacks (Giles 1987b; Milinski 1985). One hypothesis to explain this is that an increased state of hunger, caused by the plerocercoid’s nutrient theft, drives infected sticklebacks to become more active foragers (Hafer and Milinski 2016). Hafer and Milinski (2016) found that three days of starvation changed the behaviours of uninfected fish to resemble that of infected fish. We found no evidence of such an effect. Foraging and exploration behaviours increased in infected sticklebacks but were unrelated to whether fish were satiated or not. The discrepancy between our study and that of Hafer and Milinski (2016) can have several explanations, including regional differences (Heins and Baker 2008; Macnab et al. 2009) and differences in the experimental set-up. It is possible that our uninfected fish would have been bolder if we had starved them for longer than one day. In addition, we were unfortunate to only get a small number of uninfected fish in this experiment, and had a reduced statistical power to detect hunger-induced boldness among the uninfected fish. However, the fact remains that our *infected* fish were bold foragers and explorers *also* when they had just been fed to satiation. It therefore seems unlikely that the mechanism behind the well-known boldness in infected sticklebacks is merely due to hunger caused by the parasite’s nutrient theft. If there is an abundance of food, as we believe is the case in Kalmar Sound in the summer, sticklebacks are unlikely to starve for several days. Furthermore, even sticklebacks harbouring a growing plerocercoid are known to be able to grow at a normal rate and remain in good condition (Barber et al. 2008). Taken together, the effect of *S. solidus* on stickleback behaviour appears to be more specific than mere lethargy due to disease, or boldness caused by hunger (Cezilly et al. 2010; Hafer and Milinski 2016).

Experiments using recently caught fish can be seen as an ecologically more relevant approach than those using laboratory-bred fish that lack experience of, for example, real predators. However, the use of naturally infected fish has an issue of unresolved causality. For example, bolder sticklebacks may be more likely to contract infections. Several studies have now used experimental *S. solidus* infections to determine this causality and to demonstrate that it is indeed infection that causes the behavioural deviation in sticklebacks, and not vice versa (Aeschlimann et al. 2000; Barber et al. 2004; Hafer and Milinski 2016; Macnab and Barber 2012; Talarico et al. 2017). Given the general similarity of our results and those from experimentally infected sticklebacks, we believe our results are sound despite their correlative nature. It would of course be interesting to repeat our experiments with experimentally infected fish—ideally by tracking the change in behaviour as the plerocercoid grows (Barber et al. 2004). However, with experimentally infected fish there may instead be problems with habituation to the laboratory environment and with the thorny issue of how to treat the exposed-but-uninfected group in the analyses (Barber and Svensson 2003; Franke et al. 2017).

The infected fish in our study could best be described as moving around the tanks slowly but continuously. They responded to, but were relatively unaffected by, disturbances, including the exposure to perch odour. It is likely that this odd behaviour makes infected sticklebacks easier prey also for fish predators (Jakobsen et al. 1988; Milinski 1985; Ness and Foster 1999). The apparent lack of precision is interesting, given the long evolutionary history of *S. solidus* which is specific to three-spined sticklebacks (Nishimura et al. 2011), and that fish predation would be a truly disastrous outcome for this parasite (Barber and Huntingford 1995). However, a neurologically advanced manipulation of antipredator behaviours may be a complicated evolutionary feat, or carry considerable costs (Poulin 1994). Furthermore, a precise manipulation may not be required, as long as the probability of encountering the correct predators is sufficiently high (Barber et al. 2000; Milinski 1985; Parker et al. 2009). An adaptive parasite strategy could be to induce general fearlessness in the host combined with a shift in its preference for some important environmental variable, which places the host in the path of the target predators. For example, snails (*Potamopyrgus antipodarum*) infected with *Microphallus* trematodes move to the top of rocks at the time of day when the target predator is active, but retreats to cover when dead-end predators are foraging (Levri 1998). It is in fact possible that something similar occurs in our stickleback population and several lines of indirect evidence support this idea.

First, *S. solidus* plerocercoids are known to affect the stickleback’s choice of habitat (Barber et al. 2000). When the plerocercoid reaches maturity, sticklebacks are known to shift from preferring colder to preferring warmer water (Macnab and Barber 2012), a preference that benefits the growth of the plerocercoid (Franke et al. 2017). Infected sticklebacks also have a higher oxygen demand which drives them toward oxygen-rich waters (Giles 1983; Lester 1971; Meakins and Walkey 1975), and laboratory experiments have shown infected sticklebacks to spend more time close to the surface (Talarico et al. 2017). In Norwegian lakes, infected sticklebacks move to the littoral zone (Jakobsen et al. 1988), and in Alaska, infected sticklebacks prefer shallower water (Quinn et al. 2012). Giles (1987a) suggested that infected sticklebacks would seek the highest oxygen availability and this would lead them to the shore, where they were exposed to heron predation.

Second, we found a high prevalence of *S. solidus* infection in our in-shore sample, similar to other areas of the Baltic Sea, such as Poland (Macat et al. 2015) and Finland (Budria and Candolin 2015), but no infected fish further from the shore. Most of our infected fish carried only a single large plerocercoid, while multiple infections are the norm in other places (Arme and Owen 1967; Heins and Baker 2011; Heins et al. 2010b; Quinn et al. 2012). Already established plerocercoids do not seem to prevent additional infections (Arme and Owen 1967; Heins et al. 2010b; Jager and Schjorring 2006). Therefore, if 71% of our sticklebacks had at least one plerocercoid, we could expect from simple probability that around 50% (i.e. 0.71^2^) should carry two parasites, that 37% (i.e. 0.71^3^) should carry three, and so on, which was clearly not the case. Instead, the pattern with few multiple infections can be explained if the *overall* prevalence in our area is fairly low, but that infected sticklebacks aggregate in the shallow areas where we did our in-shore collection.

Third, we found no small plerocercoids (i.e. no recent infections), which is similar to a study in Alaska where most sticklebacks contracted their infections within the first few months of their lives but rarely during the following spring (Heins et al. 2016). The lack of recent infections suggests that sticklebacks with mature plerocercoids are attracted to the shore, rather than shore-loving sticklebacks being more exposed to infection.

Cezilly et al. (2010) suggested that the modification of microhabitat preferences should be a very efficient way for a parasite to reach its goal of being eaten by the correct predator, and more likely to evolve than some highly precise neurological manipulation aimed at a certain type of predation. The preference for warmer and shallower water (Franke et al. 2017; Macnab and Barber 2012; Talarico et al. 2017) should expose infected sticklebacks to herons, gulls and terns. The Kalmar area has several colonies of these birds, and black-headed gulls (*Chroicocephalus ridibundus*, L) can be seen fishing for sticklebacks by catching individuals very close to the surface (own observations). At the same time, large fish predators such as perch and cod (*Gadus morhua*, L) are unlikely to forage in very shallow areas (Prof. Per Larsson Linnaeus University pers. com.). Laboratory studies of escape behaviours suggest that *S. solidus*-infected sticklebacks are indeed easier targets (Blake et al. 2006; Jolles et al. 2020), but so far no field studies have shown birds to have greater success when attacking infected sticklebacks.

Many authors have called for caution before claiming that behavioural deviations in infected individuals are due to an active and adaptive manipulation by the parasite (Poulin 1995). First, one needs to rule out reversed causality: namely that individuals with deviating behaviours are more likely to become infected. In the case of *S. solidus*, experimental infections have repeatedly ruled out this explanation (Aeschlimann et al. 2000; Barber and Svensson 2003; Hafer and Milinski 2016; Macnab and Barber 2012; Talarico et al. 2017). Second, the case for manipulation is strengthened if the host’s behavioural change coincides with onset of infectivity, and also this has been shown for *S. solidus* (Barber et al. 2004; Macnab and Barber 2012). Third, the behavioural change should ideally increase vulnerability for the correct predator. Our results do not give unequivocal support for this. However, infected sticklebacks also appear to prefer shallow areas, where they may be preferentially exposed to the correct predators. Taken together, we suggest that *S. solidus* increases its chances of trophic transmission to birds by actively reducing anti-predator behaviours in its stickleback host. The mechanism for this seems to be to induce a general fearlessness in combination with a preference for warmer, shallower water. Even in the absence of a highly precise behavioural manipulation, *S. solidus* plerocercoids may therefore achieve parasite increased trophic transmission (PITT) by being eaten by birds. However, the critical assumption from Poulin (1995), namely that the changes in host behaviour must increase the fitness of the parasite in the field, remains to be tested.

## Financial support

This work was supported by the Faculty for Life and Health Science at Linnaeus University. It received no specific grant from any funding agency, commercial or not-for-profit sectors.

## Conflict of interest

No competing interests.

## Supplementary Information

Below is the link to the electronic supplementary material.

Supplementary file1 (DOCX 592 KB)
