# Supplementary material for: How cunning is the puppet-master? Cestode-infected fish appear generally fearless
Source: Parasitol Res. 2022 Mar 21;121(5):1305–15. doi: 10.1007/s00436-022-07470-2 (PMC8993785; doi:10.1007/s00436-022-07470-2)
Supplement: Supplementary file 1 — Supplementary file1 (DOCX 592 KB) [file 436_2022_7470_MOESM1_ESM.docx]

Supplementary Material

Svensson PS, Eriksson R, Eghbal R, Nilsson E

How cunning is the puppet-master? Cestode-infected fish appear generally fearless.

____________________________________________________________________________________

**Behavioural test arenas**

1


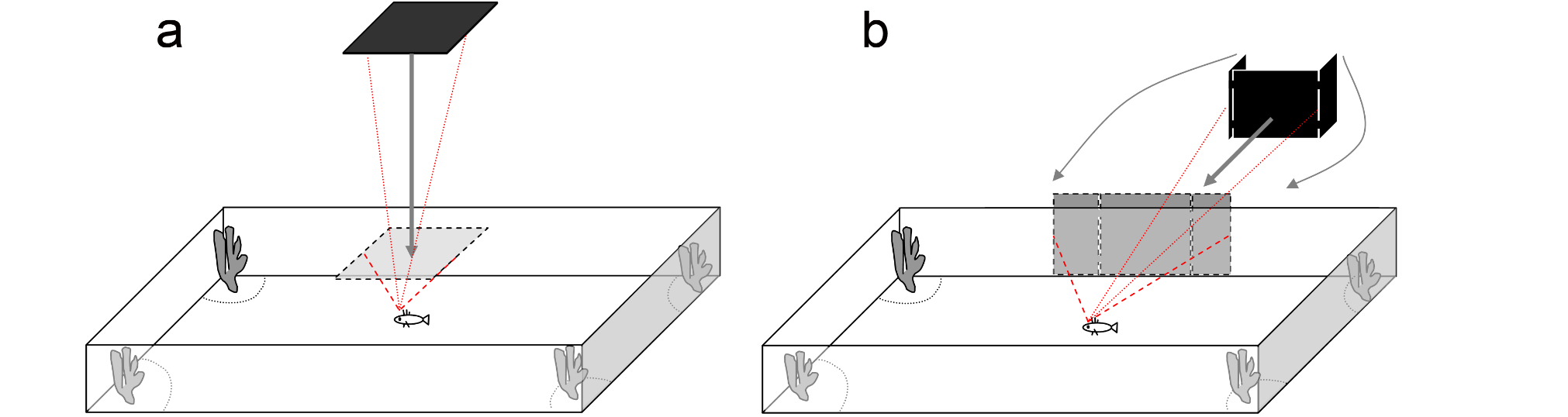


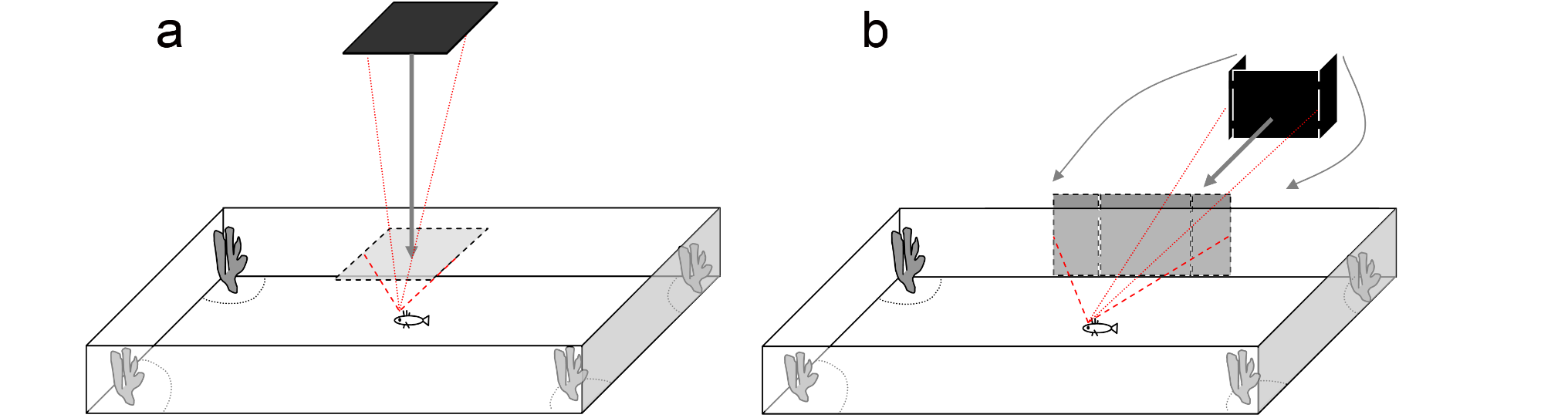


Fig SI. 1. Experimental setup for quantifying behavioural responses to simulated attacks. Sticklebacks were allowed to settle in the middle of an arena and were then exposed to one of two stimuli: a) overhead attack, where a black plate was dropped from above the arena, or b) lateral attack, where an expanding black plate quickly approached from the side. See main text for details


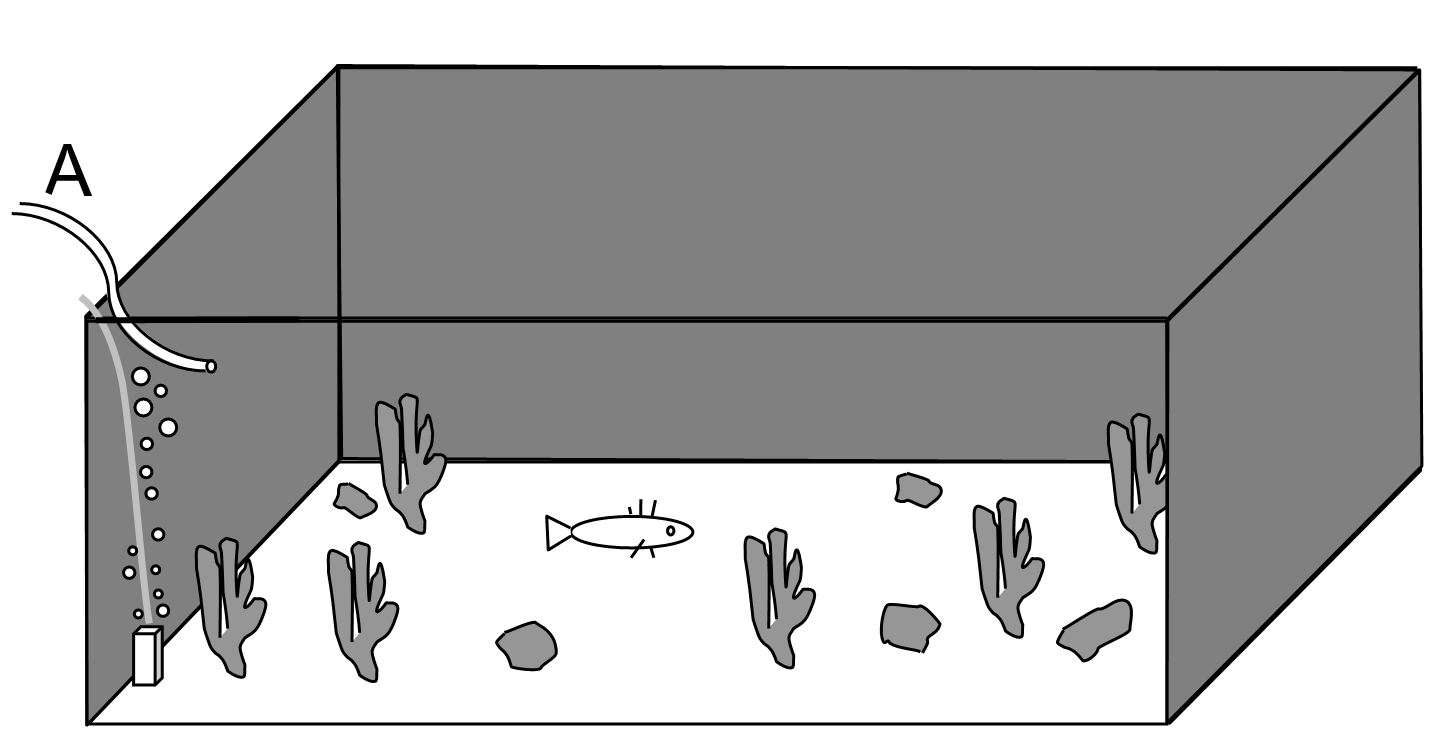


21

Fig SI. 2. Experimental setup for quantifying how sticklebacks responded to predator odour. A fish was allowed to settle in a tank with an airstone and with rocks and artificial plants as shelter. A small amount of water was then added to the tank via a hose (A). This was either pure sea water, or water from a tank with an adult perch. We then quantified the proportion of time the stickleback had their dorsal spines erected
________________________________________________________________________________________


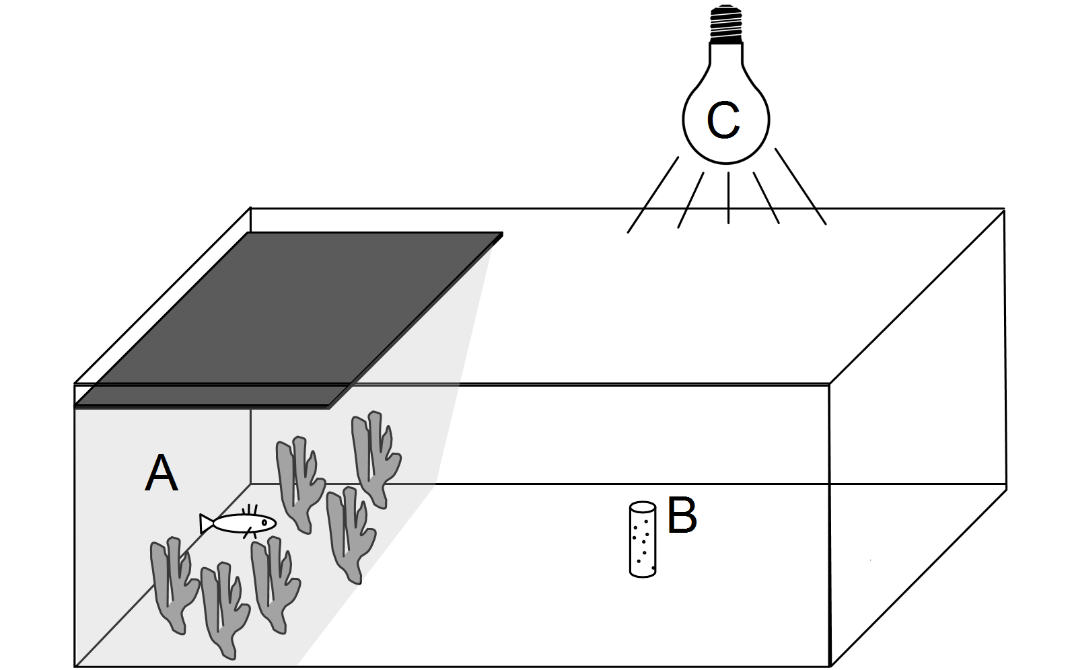


31

Fig SI. 3. Experimental setup for quantifying risk taking and foraging. Sticklebacks were released into a shaded area with several plastic plants as shelter (A). A vial with live food (B) was placed under a powerful spotlight (C). See main text for details

41


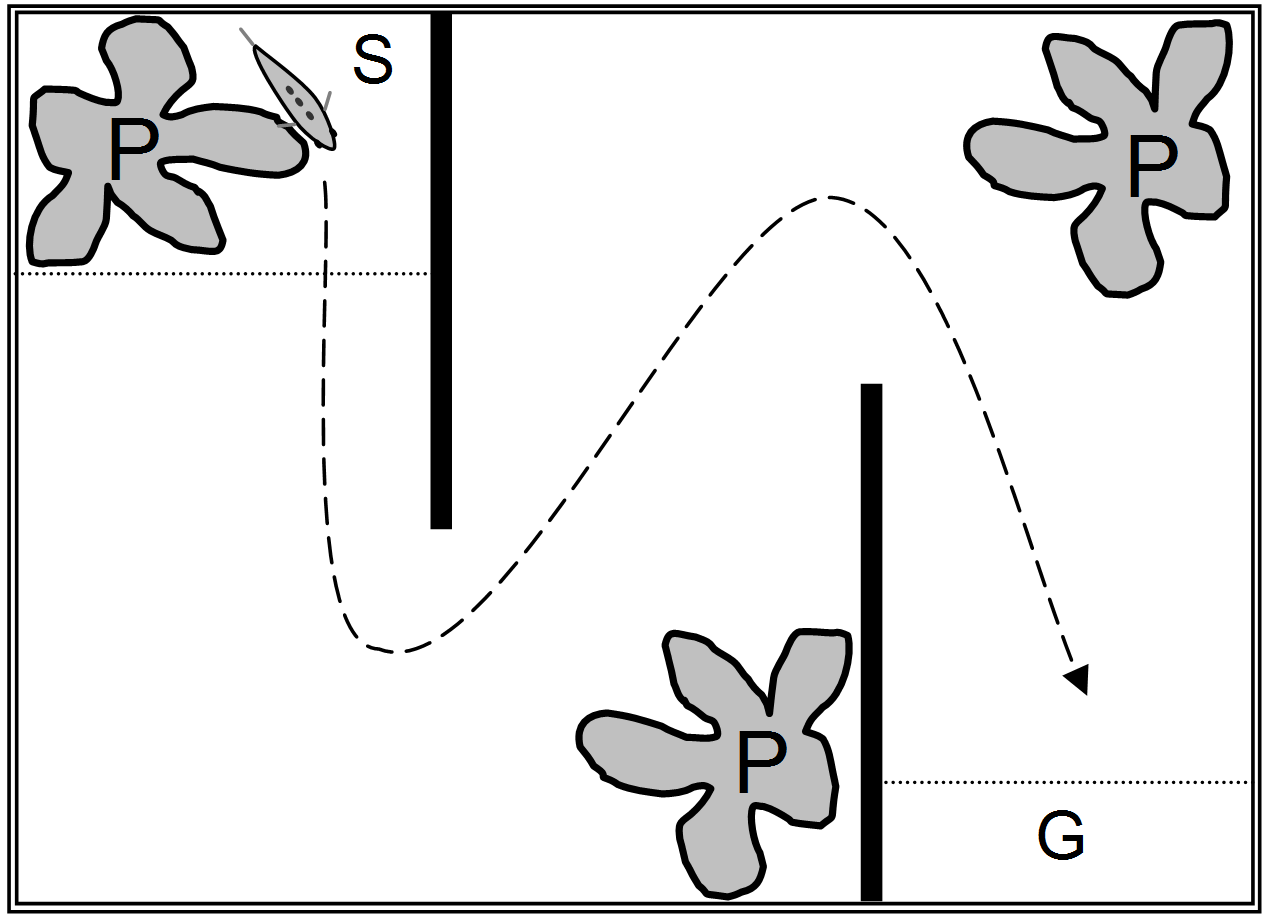


Fig SI. 4. Experimental setup for quantifying exploration behaviour (birds-eye view). Three plastic plants (P) were provided as shelter. Sticklebacks were released in the start area of the tank (S). We measured the time until the fish had left the start area and when it had explored the entire tank (i.e. reached the goal area, G). See main text for details
